# Venoarterial extracorporeal membrane oxygenation for vasoplegic shock after treprostinil refill of an implanted intravenous pump: a case report

**DOI:** 10.3389/fcvm.2024.1348311

**Published:** 2024-01-26

**Authors:** Lucía Valencia, Sergio López, Ana Olivas, Ángel Becerra, María Desirée Alemán-Segura, Marta Évora-García, Nazario Ojeda, Leonardo Cabrera, Aurelio Rodríguez-Pérez, Gregorio Pérez-Peñate

**Affiliations:** ^1^ Department of Anesthesiology and Intensive Care, Hospital Universitario de Gran Canaria Doctor Negrín, Las Palmas de Gran Canaria, Spain; ^2^ Department of Medical and Surgical Sciences, University of Las Palmas de Gran Canaria, Gran Canaria, Spain; ^3^ Multidisciplinary Pulmonary Vascular Unit, Department of Respiratory Medicine, Hospital Universitario Gran Canaria Dr. Negrín, Las Palmas de Gran Canaria, Spain; ^4^ CIBER de Enfermedades Respiratorias, Instituto de Salud Carlos III, Madrid, Spain

**Keywords:** VA-ECMO, overdose, treprostinil, vasoplegic shock, case report

## Abstract

**Introduction:**

Venoarterial extracorporeal membrane oxygenation (ECMO) is a rescue therapy that can stabilize patients with hemodynamic compromise. Indications continue to evolve, including drug overdose. However, the indication merely for vasoplegic shock following drug overdose is controversial.

**Case summary:**

We report a case of a 57-year-old male with high-risk idiopathic pulmonary arterial hypertension treated with upfront triple combination therapy (sildenafil, bosentan, and intravenous treprostinil infusion via subcutaneous abdominal implantable pump). In one of the refills of the drug reservoir, accidental administration of 1 months's supply of treprostinil (200 mg) into the subcutaneous tissue occurred, causing refractory vasoplegic shock. He required urgent VA-ECMO for 96 h, surviving to discharge 28 days later.

**Discussion:**

Treprostinil poisoning is rare due to its less frequent use but is life-threatening. ECMO may be considered in vasoplegic shock due to overdose of vasodilatory medication. It allows organ perfusion to be maintained, with the knowledge that recovery is as rapid as drug elimination.

## Introduction

Pulmonary arterial hypertension (PAH) remains a challenging condition to treat. In patients with high-risk status according to expected 1-year mortality, triple combination therapy can stabilize a patient's condition (1) and avoid lung transplantation which has a 5-year mortality of 41% even in proficient centers (2).

Triple therapy for PAH pulmonary vasodilator treatment target 3 potent mediators of vascular tone: nitric oxide, prostacyclin and endotelin pathway. The prostacyclin metabolic pathway is dysregulated in patients with PAH. Prostacyclin analogues and prostacyclin receptor agonists inhibit platelet aggregation and induce potent vasodilation. The most common adverse events observed are headache, flushing, jaw pain, and diarrhea.

Treprostinil is a prostacyclin analog (PGI2) used for the treatment of group 1 PAH. Treprostinil is available for subcutaneous, intravenous, inhaled, and oral administration. Inhaled or oral are not approved in Europe. Subcutaneous treprostinil improves exercise capacity and right heart function (3). However, on many occasions, the therapy is interrupted due to infusion-site pain.

Intravenous treprostinil can be an alternative for this adverse effect and may also be administered via implantable pumps, decreasing the occurrence of line infections (4). The intravenous implanted treprostinil pump comprises a drug reservoir, covered by a silicone septum for percutaneous refilling and a central venous catheter which is tunneled into the jugular vein. After implantation, treprostinil solution is injected monthly into the drug reservoir under aseptic conditions and the pump mechanism provides a constant intravenous flow. Treprostinil overdose is rare but can be catastrophic.

## Case presentation

**Figure 1:**
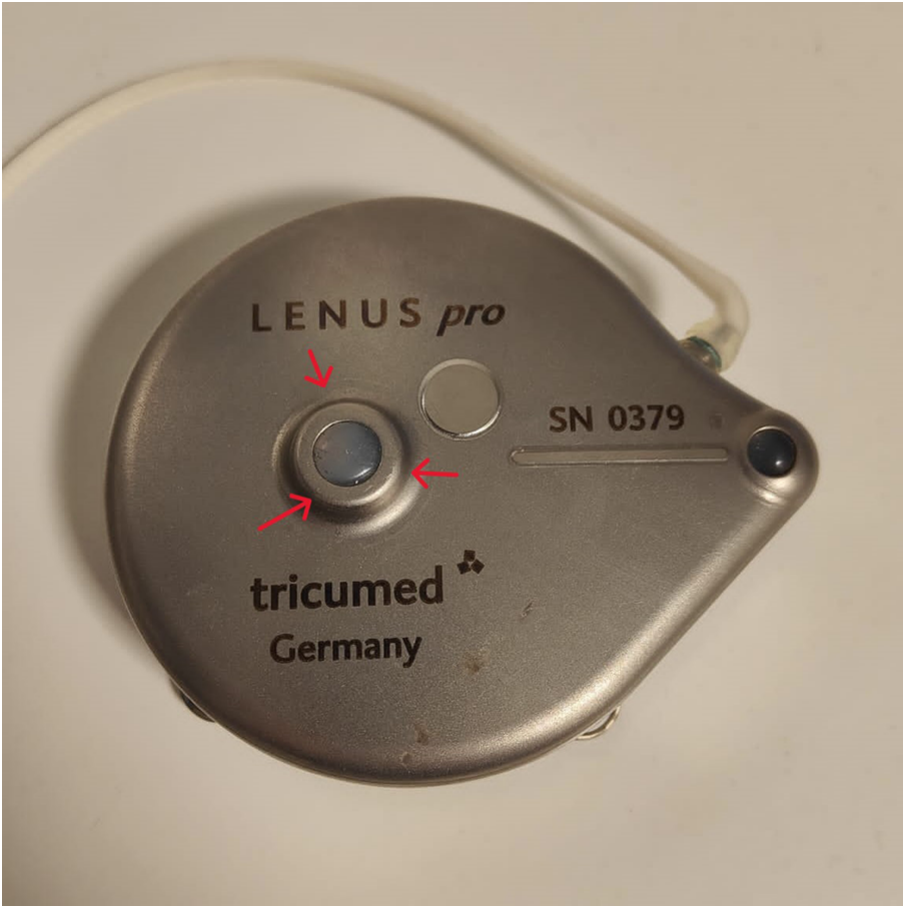
Photograph of intravenous implanted treprostinil pump. The silicone membrane where treprostinil is injected is marked with arrows.

The authors report a case involving a 57-year-old male, weight 85 kg, height 185 cm, with paroxysmal atrial fibrillation, obstructive sleep apnea, and high-risk idiopathic PAH diagnosed 5 years previously and treated with upfront triple combination therapy (sildenafil [40 mg/8 h], bosentan [125 mg/12 h], and intravenous treprostinil infusion [50 ng/kg/min] via subcutaneous abdominal implantable pump). Initially, treprostinil infusion was started subcutaneously with an external pump. Due to pain at the infusion site and repeated infections, intravenous infusion was carried out by subcutaneous implantation of the pump in the abdomen. The patient came to our hospital every 28 days to fill the pump reservoir for two years. A nurse from the Pulmonary Vascular Unit trained to fill the pump in a sterile environment proceeded to manually locate the silicone port on the top of the device (Figure 1). To ensure that the medication was injected into the reservoir, the silicone membrane was punctured, and the medication residue was removed. Once the needle position was fixed, the reservoir was progressively filled, with repeated aspirations to guarantee that the needle remained in the reservoir until the procedure was completed.

**Figure 2:**
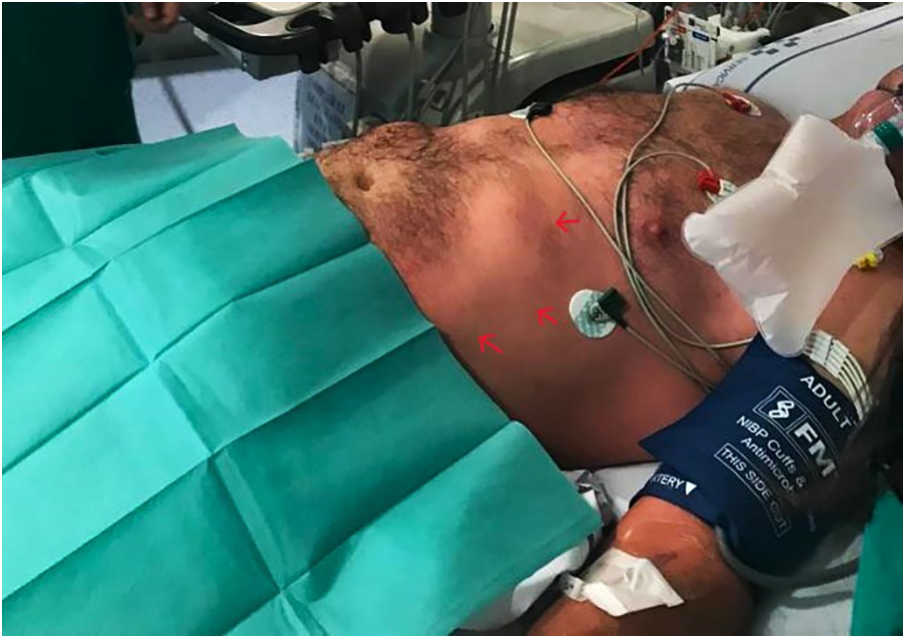
Erythematous and oedematous area around the subcutaneous pump.

In one of the refills of the drug reservoir, accidental administration of 1 months' supply of treprostinil (200 mg) into the subcutaneous tissue occurred, causing circulatory collapse. The accident probably occurred after the needle inadvertently came out of the reservoir during one of the last control aspirations. The patient was transferred to the intensive care unit and started on perfusions of norepinephrine (up to 3 mcg/kg/min) combined with epinephrine (1 mcg/kg/min) and vasopressin (0.03 IU/min). An additional 1 mg terlipressin and 1 mg/kg methylene blue were administered. Despite multiple high-dose vasopressor infusions, the patient experienced refractory shock (blood pressure 60/30 mmHg and anuria, with a serum lactate level >15 mmol/L). Chest x-ray showed bilateral alveolar infiltrates compatible with volume-overload pulmonary edema, resulting in hypoxemia (high flow nasal cannula FiO_2_ 70% for SpO_2_ 96%). Physical examination revealed generalized vasodilation, with an erythematous and edematous area around the subcutaneous pump (Figure 2). Arterial blood gas analysis showed pH: 7.29, pCO_2_: 30.8 mmol/L, pO_2_: 121 mmHg, and HCO3: 16.4.

Transthoracic echocardiography revealed hypercontractile left ventricle, with anomalous movement of the septum due to pressure overload with preserved systolic function, dilated right ventricle with preserved function. Estimated pulmonary artery systolic pressure was 60 mmHg.

**Figure 3:**
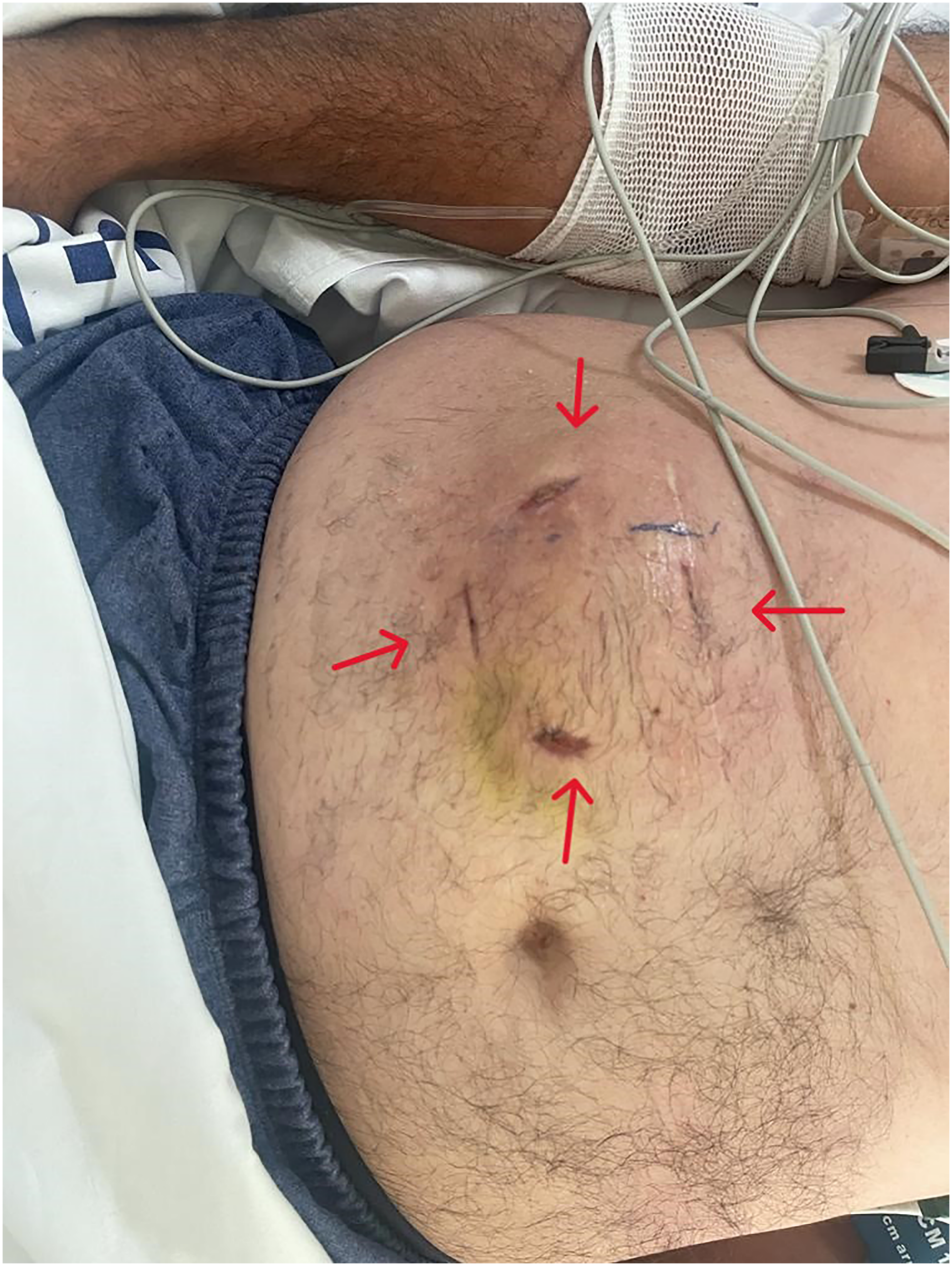
Abdominal incisions around the subcutaneous pump, 7 days later.

Attempts were made to reduce subcutaneous absorption of the drug, diluting it with peribomb injection of 100 ml of saline solution. Cold gauze was applied to cause vasoconstriction of the subcutaneous tissue and delay the absorption of treprostinil. Also, incision and drainage of the injection site was performed to remove the treprostinil, with no clinical improvement (Figure 3).

Due to refractory shock within 2 h and, given that the terminal half-life of treprostinil is 3.5 h, peripheral venoarterial extracorporeal membrane oxygenation (VA-ECMO) was planned as the rescue therapy. Cannulation was performed with semi-Seldinger technique under general anesthesia (right femoral artery cannula 19 fr × 15 cm with distal perfusion cannula 8 fr; right femoral venous cannula 23 fr × 55 cm). Pump flow was maintained at 4.5 L/min, 3,500 revolutions per minute, sweep gas flow of 4 L/min, FiO_2_ of 0.7 and activated clotting time of between 150 and 180 s. Baseline cerebral oximetry values was 76/70%, right and left side respectively, and remained within the normal range during ECMO therapy.

**Figure 4:**
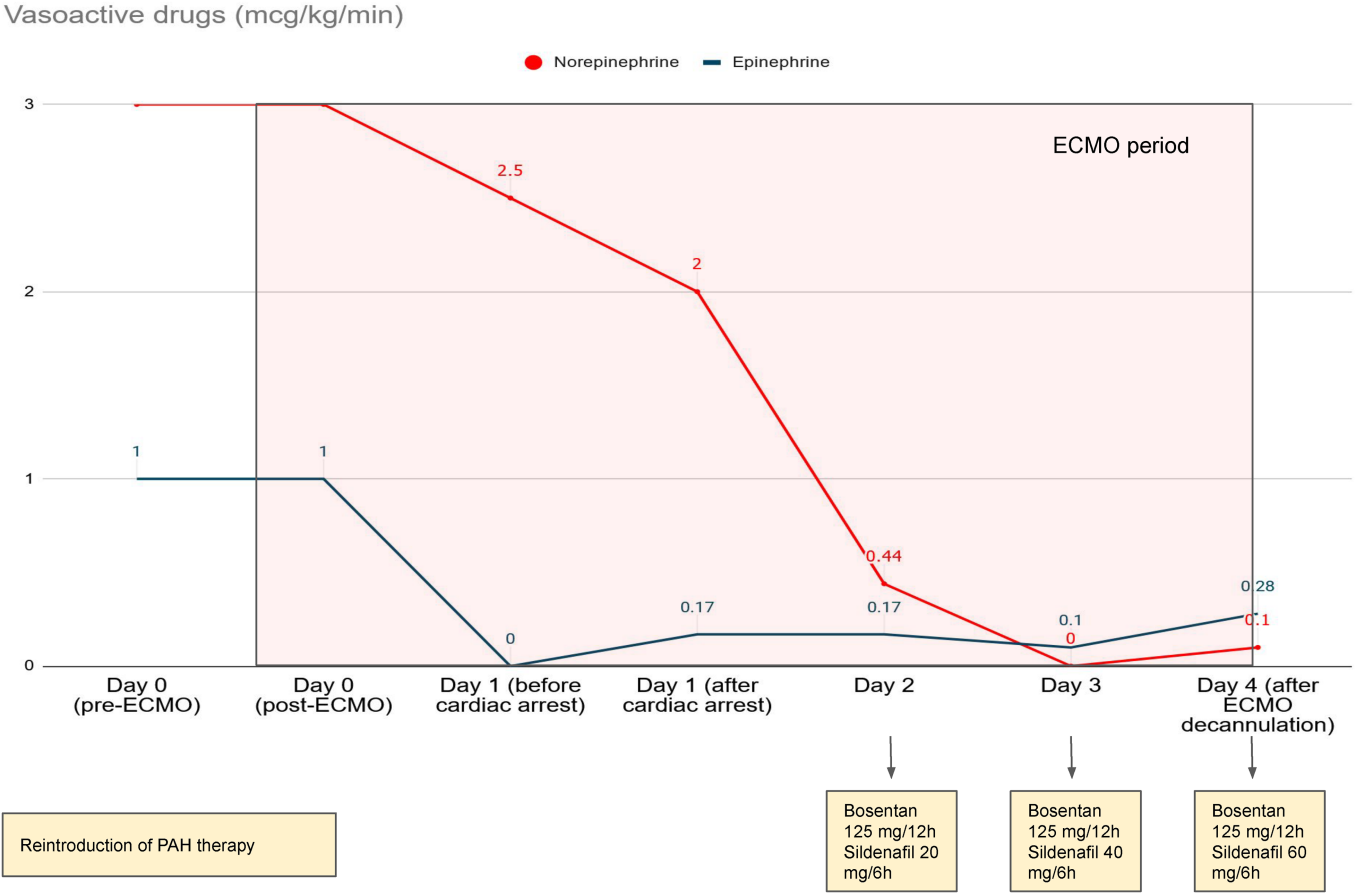
Trends in catecholamines and restart of pulmonary arterial hypertension treatment.

In the following hours, the patient's blood pressure increased to 90/60 mmHg. Improvement of indirect signs of cardiac output, with recovery of diuresis and normalization of lactacidemia (serum lactate level 1.8 mmol/L) was observed. After achieving hemodynamic stability 12 h post-ECMO, epinephrine was gradually withdrawn. Because of the decrease in inotropic drugs and the high afterload due to the retrograde flow of ECMO, pulseless electrical activity occurred. Transthoracic echocardiography revealed a dilated left ventricle with biventricular dysfunction without aortic valve opening. Promptly epinephrine was reintroduced up to 0.17 mcg/kg/min, and pump flow was decreased up to 2 lpm to reduce afterload. After 20 min of mechanical cardiopulmonary resuscitation [LUCAS (Physio-Control/Jolife AB, Lund, Sweden)], recovery of spontaneous circulation occurred. One day later, sildenafil and bosentan were gradually re-introduced (Figure 4). The patient was progressively weaned from pump, guided by echocardiogaphy, maintaining velocity–time integral (VTI) around 10 cm, which allowed for decannulation of the patient at 96 h and subsequent extubation.

Twenty-four hours after ECMO withdrawal, intravenous perfusion of treprostinil (2.38 ng/kg/min) was progressively titrated up to the initial dose. The patient was transferred to the intermediate respiratory care unit and 28 days later discharged.

As mentioned above, due to the possibility of pump malfunction, treprostinil perfusion was started via central venous catheter, given that the abdominal area was swollen after subcutaneous drainage. Subsequently, although the patient was reluctant, the subcutaneous implantable pump was restarted, initially with saline, and later with treprostinil, noting the decrease in infusion rate to 0.06 ml/day. Simultaneously, intravenous treprostinil perfusion with external pump was maintained at home for 4 months until it was switched to the implanted pump. Currently, the patient remains clinically stable, and satisfied with the pump's restart, with a good quality of life.

## Discussion

The patient described herein experienced refractory cardiocirculatory collapse and was uccessfully resuscitated using VA-ECMO even though the indication for VA-ECMO in vasoplegic shock is controversial. Vasoplegic or vasodilatory shock, is a condition defined by profound vasodilation due to persistently low systemic vascular resistance with normal or high cardiac index (5). Thus, maintaining adequate ECMO flows in this group of patients may be difficult due to relative intravascular hypovolemia. Consequently, the role of ECMO may be challenging in this condition.

Vasoplegic shock includes multiple and diverse etiologies, the most common types of this shock are septic, anaphylactic, and drug overdose. Numerous cases of septic shock in which ECMO is indicated have been reported since 2013, with a low survival rate of around 15% (6). Since then, single-center studies have emerged with a 36% improvement in survival. These studies were evaluated in a recent metanalysis of 468 patients, in which survival was higher in patients with EF <20% compared to those with EF >35% (62% vs. 32.1%) (7). Sepsis-induced cardiomyopathy is an increasingly recognized entity characterized by myocardial dysfunction in a sepsis setting. Up to half of patients with septic shock demonstrate some level of sepsis induced cardiomyopathy. The most recent Surviving Sepsis Campaign International Guidelines in 2021 do not include recommendations for VA-ECMO in cardiomyopathy refractory to inotropes. Considering all this data, the experts concluded VA-ECMO might be a reasonable treatment option in refractory septic shock when cardiac dysfunction is associated (8).

Anaphylactic shock is also characterized by arteriolar vasodilation resulting in vasoplegic shock. Additionally, in the context of most severe anaphylactic shock requiring the need for ECMO, myocardial function is impaired. Sometimes, Kounis syndrome (coincidental occurrence of an acute coronary syndrome with hypersensitivity reactions following an allergic event) may cause myocardial dysfunction (9). There are various case reports of successful use of ECMO for anaphylactic shock (10). All of them involved severe allergic reactions, with left ventricular dysfunction (11–14), and some of them include Kounis syndrome (9, 15).

Another type of vasoplegic shock in which ECMO may be indicated is drug overdose, as in our patient. Other documented cases of VA-ECMO rescue for overdose such as amlodipine (16, 17), and metformin have been published (18). Overdose of both drugs not only cause vasoplegic shock but also cardiogenic shock. In metformin poisoning, intermittent hemodialysis or continuous renal replacement therapy is the first therapeutic rescue, but VA-ECMO support should be performed immediately when cardiac function is significantly depressed (18). On the other hand, amlodipine acts by blocking the voltage-sensitive (l-type) calcium channels 3 and thus affects not only vascular smooth muscle tone, but also myocardial contractility, automaticity, and atrioventricular conduction.

Unlike other intoxications, treprostinil overdose is strictly a vasoplegic shock with no cardiotoxic component. In our center we normally perform a transthoracic echocardiography prior to ECMO cannulation to confirm the choice of ECMO configuration. In this case, it allowed us to determine that biventricular function was not impaired. VA-ECMO was planned as desperate therapy.

Treprostinil poisoning is rare due to its less frequent use but is life-threatening. In adults, there are two published cases, without requiring ECMO, since the doses administered subcutaneously were lower than in our patient [100 mg subcutaneous (19) y 7.5 mg subcutaneous (20)]. The treatment was only hemodynamic support since treprostinil may not be reliably eliminated by hemodialysis, and no specific antidote is available. However, in refractory shock, even in merely vasoplegic shock, ECMO serves as a bridge-to-recovery by maintaining perfusion to vital organs, affording time for drug elimination.

Treprostinil does have a half-life of 3.5 h. Therefore, drug elimination should finish after 12–16 h. However, Hohenforst-Schmidt et al. (19) noticed patient experienced prolonged hemodynamic effects. In our case it was difficult to assess how long the vasodilator effect of treprostinil lasted, given that the patient was supported with ECMO but norepinephrine begins to decrease after 24–48 h. Predicting the absorption of a subcutaneous drug was difficult, after injecting saline into the area and placing cold gauze to delay absorption. Although this measure did not seem to be effective at first, it may have contributed to reducing the severity of the shock.

The use of VA-ECMO for vasodilatory shock carries the risk for cerebral hypoperfusion and aortic valve closure by exposing the heart to increased afterload due to high doses of alpha-adrenergic catecholamines. Optimal monitoring such as near-infrared spectroscopy, pulse oximetry saturation in the right hand, and serial transthoracic echocardiography is essential to avoid complications (21). Diary echocardiography is mandatory in VA-ECMO. Weaning trials are essential to assess the behavior of ventricles during increases in preload. Moreover, the pulmonary artery catheter could have helped in our case. When monitoring pulmonary artery pressures in a patient with VA-ECMO we must understand that they are not real since the cardiac output passing through the pulmonary artery is only 40%. Apart from the unreliability of the Swan–Ganz parameters resulting from suction of the venous ECMO cannula, some authors reject the use of a Swan–Ganz catheter in patients on VA-ECMO support due to safety issues such as migration of the catheter or introduction of air to the ECMO system (22). However, it plays an important role in weaning from ECMO, supporting the echocardiogram, since elevated capillary pressures support left ventricular failure. Moreover, in our case, the pulmonary artery monitoring would have helped us restart PAH medication more accurately.

To our knowledge, this is the first case of failure in treprostinil refill and vasoplegic catastrophic shock requiring VA-ECMO. ECMO may be considered in vasoplegic shock due to overdose of vasodilatory medication. It allows organ perfusion to be maintained, with the knowledge that recovery is as rapid as drug elimination. Continuous monitoring is necessary to determine the optimal time to wean the patient off ECMO to avoid complications, because in vasoplegic shock, the risk of cerebral hypoperfusion and aortic valve closure is higher than in other indications for VA ECMO.

Finally, emphasizing and reinforcing the safety of monthly pump refill is critical. In cases of difficulty in locating the entry port, ultrasound should be used to ensure the initial position of the needle. Also, it is very important that the filling of the pump is done progressively with small strokes followed by aspirations to ensure that the needle remains in the reservoir throughout the procedure. We highlight the slowing of pump infusion rate after its temporary cessation.

## Data Availability

The original contributions presented in the study are included in the article/Supplementary Material, further inquiries can be directed to the corresponding authors.
